# *N*-Acetyl-Cysteine Regenerates Albumin Cys34 by a Thiol-Disulfide Breaking Mechanism: An Explanation of Its Extracellular Antioxidant Activity

**DOI:** 10.3390/antiox9050367

**Published:** 2020-04-28

**Authors:** Alessandra Altomare, Giovanna Baron, Maura Brioschi, Martina Longoni, Riccardo Butti, Edoardo Valvassori, Elena Tremoli, Marina Carini, Piergiuseppe Agostoni, Giulio Vistoli, Cristina Banfi, Giancarlo Aldini

**Affiliations:** 1 Department of Pharmaceutical Sciences (DISFARM), Università degli Studi di Milano, Via Mangiagalli 25, 20133 Milan, Italy; giovanna.baron@unimi.it (G.B.); martinalongoni@yahoo.it (M.L.); riccardo.butti@studenti.unimi.it (R.B.); edoardo.valvassori@gmail.com (E.V.); marina.carini@unimi.it (M.C.); giulio.vistoli@unimi.it (G.V.); giancarlo.aldini@unimi.it (G.A.); 2 Centro Cardiologico Monzino, Istituti di Ricovero e Cura a Carattere Scientifico (IRCCS), Via Parea 4, 20138 Milan, Italy; maura.brioschi@cardiologicomonzino.it (M.B.); elena.tremoli@cardiologicomonzino.it (E.T.); piergiuseppe.agostoni@cardiologicomonzino.it (P.A.); cristina.banfi@cardiologicomonzino.it (C.B.); 3 Department of Clinical Sciences and Community Health, Cardiovascular Section, Università degli Studi di Milano, Via della Commenda 19, 20122 Milan, Italy

**Keywords:** *N*-acetyl-cysteine, albumin, Cys34, cysteine, antioxidant, plasma

## Abstract

In the present paper, the extracellular antioxidant activity of *N*-acetyl-cysteine (NAC) is explained by considering its ability to regenerate the free form of albumin Cys34 by breaking the disulfide bond of the cysteinylated form (HSA-Cys). NAC’s capability to regenerate albumin Cys34 (HSA-SH) was studied by MS intact protein analysis in human plasma and in a concentration range of NAC easily achievable after oral and i.v. administration (5–50 µg/mL). NAC dose-dependently broke the HSA-Cys bond to form the dimer NAC-Cys thus regenerating Cys34, whose reduced state was maintained for at least 120 min. Cys was faster in restoring Cys34, according to the reaction constant determined with the glutathione disulfide (GSSG) reaction, but after 60 min the mixed disulfide HSA-Cys turned back due to the reaction of the dimer Cys-Cys with Cys34. The explanation for the different rate exchanges between Cys-Cys and Cys-NAC with Cys34 was given by molecular modeling studies. Finally, the Cys34 regenerating effect of NAC was related to its ability to improve the total antioxidant capacity of plasma (TRAP assay). The results well indicate that NAC greatly increases the plasma antioxidant activity and this effect is not reached by a direct effect but through the regenerating effect of Cys34.

## 1. Introduction

*N*-acetylcysteine (NAC), the *N*-acetyl derivative of the natural amino acid l-cysteine, is a reference antioxidant compound widely used in in vitro and ex vivo conditions. The antioxidant activity of NAC is attributed to the thiol group in the thiolate form which acts by scavenging radical and non-radical oxidants (direct effect) as well to the fact that it acts as a precursor of Cys (through a deacetylation reaction catalyzed by aminoacylase I) which is the building block of glutathione, GSH (indirect effect), the main intracellular antioxidant and co-factor of several antioxidant and detoxifying enzymes [1,2].

A critical insight into the antioxidant property of NAC showed that the direct antioxidant activity of NAC is not really efficient, in particular when compared to other endogenous thiols such as Cys or GSH [1]. This is explained by the low acidity of the NAC thiol as a consequence of the amino group acetylation, making the equilibrium between the antioxidant form (thiolate) and the thiol, largely shift toward the latter, which is the inactive from. Taking into account the pKa of the thiol group of NAC (pKa = 9.5), at. pH 7.4, the fraction of the active form is 0.76/100 with respect to the non-active form, while for Cys (pKa = 8.6) and GSH (pKa = 9.2), it is 6.3/100 and 1.58/100, respectively. On the basis of these data, we can calculate that at a fixed concentration, the active forms (thiolate) of GSH and Cys are almost two and eight fold if compared to NAC, respectively. By considering the reaction rates of the endogenous antioxidants, of the substrates and of NAC toward the main radical and non-radical oxidants, it can be assumed that NAC acts as a direct antioxidant agent only toward some specific oxidants and when it is present in a high concentration as when it occurs in in vitro condition. 

Based on these premises, we can assume that in in vivo conditions, NAC performs as a cellular antioxidant by acting as a precursor of GSH but it is still not clear whether and how it works as an extracellular antioxidant where the content of GSH is quite negligible. 

Cys34 of albumin represents the main extracellular antioxidant, characterized by a much higher antioxidant activity with respect to that of Cys and NAC due to its peculiar acidity (pKa = 8.1) which is given by a particular microenvironment able to stabilize the thiolate anion [3]. When Cys34 acts as an antioxidant, the corresponding sulfenic acid derivative is formed, which then reacts with free Cys (the main extracellular low molecular thiol) forming the corresponding mixed disulfide (cysteinylation reaction) thus preserving the irreversible oxidation of Cys34 to sulfinic and sulfonic acid derivatives [4]. The cysteinylated form of Cys34 significantly increases in several physio-pathological oxidative conditions such as aging [5], intrauterine growth restriction [6], cirrhosis [7], and renal disease [8]. In particular, the physiological content of the cysteinylated form in healthy subjects is <30%, increasing to ≥40% in oxidative stress conditions [9]. Although it is not yet clear which is the endogenous pathway deputed to restore Cys34 from the corresponding cysteinylated form, it was found that NAC is able to restore albumin Cys34 by breaking the cysteinylated form [10]. In this reaction a not effective direct antioxidant (NAC) is consumed to regenerate the highly effective Cys34, thus making NAC act as an indirect antioxidant activity.

The beneficial role of Cys34 is not only be related to the extracellular antioxidant but also to its ability to detoxify several electrophilic and toxic agents [11] such as 4-hydroxynonenal which is involved in several diseases including metabolic disorders [12,13,14], and cardiovascular diseases [15]. Considering that the conversion of Cys34 to the cysteinylated form significantly increases in several oxidative-based diseases, we believe that the ability of NAC to selectively restore Cys34 can have a pivotal role in maintaining the antioxidant and electrophilic detoxification potential of the extracellular milieu. Hence NAC is an old molecule which can exert a very promising therapeutic potential through a mechanism of action which merits deeper investigation.

The aim of this paper is to better characterize the reaction mechanism of NAC as a restoring agent of Cys34, to compare the reaction rate with respect to that of Cys, and to correlate the thiol-breaking activity of NAC to its antioxidant activity in human plasma. To reach this goal we firstly examined the thiol-disulfide breaking activity of NAC in in vitro conditions using GSSG and cysteinylated albumin as substrates and Cys as reference compound. The ability of NAC to regenerate albumin Cys34 was then confirmed in human plasma and in a concentration range of NAC easily reachable after oral and iv administration and using human plasma from healthy donors. Finally, the Cys34 regenerating effect of NAC was related to its ability to improve the total antioxidant capacity of plasma as measured by the TRAP assay. The results well indicate that NAC, at a concentration range which can be easily reached after a single oral or iv administration, greatly increases the plasma antioxidant activity and this effect is not reached by a direct effect but through a regenerating effect of Cys34 through a thiol-disulfide breaking mechanism of the cysteinylated albumin. Cys, even if more effective as a thiol-disulfide breaking agent, was much less active as antioxidant because it is less effective in maintaining the Cys34 in a free form due to the reaction of cystine with Cys34.

## 2. Materials and Methods 

### 2.1. Reagents

Formic acid (FA), acetonitrile (CH_3_CN) and 2,2,3,3,4,4,5,5,5-nonafluoropentanoic acid (NFPA) were of liquid chromatography-mass spectrometry (LC-MS) grade; *N*-Acetyl-l-cysteine (NAC), cysteine (Cys), l-Glutathione oxidized (GSSG), human plasma from human male AB, hydrogen peroxide solution 30% (w/w) in H_2_O, NaCl, sodium dihydrophosphate, disodium phosphate, 2’,7’-Dichlorofluorescin diacetate (DCFH-DA), 2,2’-Azobis(2-amidinopropane) dihydrochloride (AAPH) and all other chemicals were analytical grade and purchased from Sigma-Aldrich (Milan, Italy). Ultrapure water was prepared by a Milli-Q purification system (Millipore, Bedford, MA, USA). 

Plasma samples were obtained from a subset of healthy subjects (controls) from a previously enrolled population. The study was approved by the Ethical Committee European Institute of Oncology and Monzino Cardiologic Center (registration number R391/16-CCM406).

### 2.2. GSSG Disulfides Breaking Activity of NAC and Cys

The rate constants of the thiol-disulfide exchange reaction mediated by NAC and Cys were measured by using GSSG as disulfide. The time-dependent disappearance of GSSG and the formation of GSH and of the disulfides (GSH-NAC, GSH-Cys) were determined by LC-ESI-MS/MS. The incubation mixtures were prepared in 100 mM phosphate buffer at pH 7.4 using the following stoichiometric ratios between GSSG and the thiol: 1:3–1:2–1:1–1:0.5 and maintaining the GSSG concentration constant (5 mM). The mixtures were incubated at 37 °C under slow stirring (300 RPM) in the Thermomixer (Eppendorf). Aliquots of the reaction mixtures were taken at different time points (T_min_: T_0_, T_1_, T_2_, T_3_, T_4_, T_5_, T_7.5_, T_10_ and T_15_), diluted 1:500 with H_2_O:CH_3_CN:NFPA 95:5:0.08% v:v:v, filtered using 0.45 μm Ø13 mm PVDF filters and injected in triplicate in HPLC-MS (10 μL). Analyses were carried out using a LC-ESI-MS/MS method in Multiple Reaction Monitoring (MRM). The chromatographic separation was performed on a reversed-phase Phenomenex Kinetex Core-Shell technology-C18 (75 × 2.1 mm, i.d. 2.6 µm, 100 Å, Milan, Italy) protected by a Phenomenex Kinetex security-guard column, by using a quaternary pump HPLC system (Surveyor LC system, ThermoQuest, Milan, Italy) equipped with thermostated compartment for the column kept at 40 °C and an autosampler maintained at 8 °C, working at a constant flow rate (200 µL/min). Aliquots of 10 µL of the standard or sample solution were injected in partial loop mode and analytes were eluted with a 18 min multistep gradient of phase A (H_2_O:NFPA, 100:0.08% v/v) and phase B (CH_3_CN): 0–1 minutes, isocratic of 5% B; 1–3 minutes, from 5% B to 40% B; 3–7 minutes, from 40% B to 70% B; 7–12 minutes, isocratic of 60% B; 12–13 minutes, from 60% B to 95% B; 1 minute of isocratic 95% B; and then 4 minutes of isocratic 5% B. The MS/MS analyses were performed with a TSQ Quantum Triple Quadrupole (Thermo Finnigan Italy, Milan, Italy) mass spectrometer fitted with an electrospray (ESI) interface operating in positive-ion mode, under the following source parameters: capillary temperature, 275 °C; spray voltage 4.5 kV; capillary voltage, 35 V; and tube lens voltage 102 V. The parameters influencing the transitions were optimized as follows: argon gas pressure in the collision Q2, 1.0 mTorr; peak full width at half-maximum (fwhm), 0.70 *m*/*z* at Q1 and Q3; scan width for all MRM channels, 0.002 *m*/*z*; scan time 0.675 s, skimmer offset 10 V.

GSSG analysis was carried out by using the following transitions:

GSSG: *m*/*z* 613.00 → 355.00      (collision energy 15 eV)

The calibration curve for GSSG dissolved in H_2_O/CH_3_CN/NFPA 95:5:0.08% vol:vol:vol was built using a concentration range of 0.156 µM–10.000 µM. The peptide LQQCPF was used as internal standard. Carry-over effects were assessed, with relevant criteria by injecting samples followed by the calibration standard at the highest concentration. The limit of detection (LOD) was calculated using a signal to noise ratio (S/N) ≤ 3. Data processing was performed by using Xcalibur 2.0 software.

The formation of GSH and the adducts GSH-NAC and GSH-Cys disulfides was monitored by using the following transitions:

GSH: *m*/*z* 308.00 → 162.00 + 179.00 + 233.00 (collision energy 15 eV)

GSH-NAC: *m*/*z* 469.00 → 340.00     (collision energy 15 eV) 

GSH-Cys: *m*/*z* 427.10 → 177.00 + 298.00   (collision energy 20 eV)

The standards of the mixed disulfides were prepared from a reaction mixture prepared at a stoichiometric ratio of 1:3 of GSH and thiol (NAC/Cys) respectively, in 100 mM phosphate buffer at pH 7.4; the reaction mixture was incubated at 37 °C for 120 minutes, kept under slow stirring (300 RPM) using the Thermomixer (Eppendorf). H_2_O_2_ at a final concentration of 1 mM was added to catalyze the formation of the mixed disulfides. Kapp values and the pH-independent rate constants were calculated according to the method reported by Ferrer-Sueta et al. [16].

### 2.3. Albumin Cys34-Cys Breaking Activity

#### 2.3.1. Sample Preparation

The time-dependent activity of Cys and NAC in breaking cysteinylated albumin and in regenerating mercaptoalbumin was determined by using human plasma from Sigma and measuring the relative content of the two isoforms of albumin by MS intact protein analysis as already described [8]. Aliquots of 1 mL of plasma were spiked with NAC or Cys at the following final concentrations: 100, 50, 25 µg/mL (plasma); NAC and Cys stock solutions were previously prepared dissolving both reagents in 100 mM phosphate buffer at pH 7.4, at a concentration of 1 mg/mL.

The reaction mixtures were incubated at 37 °C under slow stirring (400 RPM) in the Thermomixer (Eppendorf) and at the following reaction time points (T_min_: T_0_, T_1_, T_2_, T_3_, T_5_, T_10_, T_20_, T_30_, T_60_, and T_120_) aliquots were withdrawn and diluted 1:40 with a mixture of H_2_O:CH_3_CN:FA 70:30:0.1% v:v:v to stop the reaction. The diluted solutions were transferred into the vials and injected twice (technical duplicate) into a HPLC-MS system. 

#### 2.3.2. Intact Protein Analysis by LC-Mass Spectrometry

The analyses were performed on a reversed-phase Phenomenex LC column Jupiter-C4 (150 × 2 mm, i.d. 5 µm, 300 Å, Milan, Italy) protected by a Phenomenex security-guard column, by using a Surveyor system (ThermoFinnigan Italy, Milan, Italy) equipped with thermostated compartment for the column kept at 40 °C and an autosampler at 8 °C, working at a constant flow rate (250 µL/min). 10 µL of standard solutions were injected in partial loop mode and analytes were eluted with a 20min multistep gradient of phase A H_2_O:FA (100:0.1% v:v) and organic phase B CH_3_CN:FA (100:0.1% v:v): 0–2 minutes, isocratic of 30% B; 2–13 minutes, from 30% B to 50% B; 13–14 minutes, from 50% B to 95% B; 14–17 minutes, of isocratic 95% B; and then 3 minutes of isocratic 30% B. To prevent potential signal suppression due to the presence of salts coming from the buffer used for sample preparation, during the first 3 minutes of analysis the eluted mixture was discarded by using the Divert Valve; after the first minute of analysis the valve was set in a position allowing the elution of the mixture directly in the source. The eluted species coming from the chromatographic system were analyzed by a triple quadrupole (TQ) mass spectrometer (Finnigan TSQ Quantum Ultra, ThermoQuest, Milan, Italy) equipped with an Electrospray Finnigan Ion Max source. MS spectra were acquired under the following instrumental conditions: positive-ion mode; ESI voltage 3.5 kV, capillary temperature 320 °C, capillary voltage 46 V, Q3 scan range 1200–1500 *m*/*z*, Q3 power 0.4 amu, scan time 1 s, Q2 gas pressure 1.5 Torr, skimmer offset 10 V, microscan set to 3. Full instrument control and ESI mass spectra acquisitions were carried out by Xcalibur software (version 2.0.7, Thermo Fisher Scientific, Rodano, MI, Italy). 

#### 2.3.3. Data Elaboration

Mass spectra deconvolution was performed using MagTran software (version 1.02, author: Zhongqi Zhang, Center for Interdisciplinary Magnetic Resonance, National High Magnetic Field Laboratory, Florida State University, Tallahassee, Florida, USA), implemented by an algorithm that allows the calculation of the molecular weight of an analyte based on the *m*/*z* values of the single multicharged ions recorded. In this way it was possible to monitor the relative content of the following HSA isoforms: mercaptoalbumin (HSA-SH), cysteinylated HSA (HSA-Cys), glycated HSA (HSA-gly), and other species not yet identified. Using the spectrum smoothing tool of the MagTran software, and setting the following parameters: Gaussian Deconvolution Options → Min peak width: 5 Da; Max peak width: 50 Da; Max iteration: 256; Rho: 0.75; Max no. of gaussians: 5–8, it was possible to calculate the peak area of each species and the corresponding relative abundance (%) was calculated. The data relative to the content of mercaptoalbumin and HSA in the cysteinylated form were further elaborated to calculate, for both species, at each time point, the average values, and standard deviations based on three injections. 

### 2.4. Measurement of Plasma Antioxidant Capacity

The plasma antioxidant activity in absence and presence of NAC or Cys was evaluated by a fluorimetric assay based on 2’,7’-dichlorodihydrofluorescein diacetate (DCFH_2_-DA) as substrate and AAPH as radical initiator [16].

The DCFH_2_ was prepared starting from 2’,7’-dichlorodihydrofluorescein diacetate (DCFH_2_-DA) by basic hydrolysis: briefly 500 μL of DCFH-DA stock solution (1 mM) was mixed with 2 mL of NaOH (0.01 N at 4 °C) for 20 min while protected from the light. The mixture was then neutralized with 2 mL of HCl (0.01 N), diluted with phosphate-buffered saline (PBS) to a final concentration of 10 μM and stored in ice for no longer than 8 h (working solution); 

NAC and Cys stock solutions were prepared by dissolving the reagents in 100 mM phosphate buffer at pH 7.4, at a finale concentration of 1 mg/mL. Plasma samples in absence or presence of NAC or Cys at different concentrations (25, 50, 100 µg/µL) were incubated at 37 °C under slow stirring (400 RPM) in the Thermomixer (Eppendorf) for 30 minutes. Then 40 µL of the above mentioned samples were spiked with DCFH_2_ and AAPH at the final concentration of 10 µM and 10mM, respectively. Samples were loaded in triplicate on a 96-well plate (BRANDplates pureGrade, BRAND^®^, 97877 Wertheim, Germany) and the 2-electron oxidation of DCFH to the highly fluorescent compound 2’,7’-dichlorofluorescein (DCF) was monitored at 37 °C using a multilabel, multitask plate reader (Victor-1420 Multilabel Counter; Wallac, Turku, Finland) setting the excitation and emission wavelengths at λex 485 nm λem at 535 nm, respectively.

### 2.5. Molecular Modeling 

The computational analyses were focused on the nucleophilic exchange between the possible disulfides (Cys-Cys and Cys-NAC) and Cys34 (represented by methanethiol for simplicity) as simulated by density functional theory (DFT) calculations involving the corresponding anionic trisulfide intermediates. In detail the conformational profile of the two studied disulfides was explored by MonteCarlo procedures as implemented in the VEGA suite of programs [17] which generate 1000 conformers by randomly rotating the flexible bonds. The so obtained best structures were optimized by PM7 semi-empirical method by MOPAC [18] and used to build the corresponding trisulfide intermediates. All considered molecules were simulated at their ground-state and at gas phase by density functional theory (DFT) using the Becke three-parameter hybrid function with LYP (Lee, Yang, Parr) correlation (DFT/B3LYP) and with the 6-31G basis set as implemented by the GAMESS software [19]. To evaluate the effect of water solvents, the DFT calculations were repeated by applying the polarizable continuum model (PCM) implicit solvent model.

### 2.6. Satistical Analysis

One-way ANOVA followed by Dunnett’s multiple comparisons test was performed using GraphPad Prism version 8.0.0 for Windows (GraphPad Software, San Diego, CA USA).

## 3. Results

### 3.1. GSSG Disulfide Breaking Activity of NAC and Cys

The first step was to evaluate the thiol-disulfide breaking activity of NAC and to compare its reactivity with that of Cys, using GSSG as disulfide. The disulfide breaking activity was monitored by measuring the time-dependent consumption of GSSG as determined by LC-ESI-MS in MRM mode. NAC induced a time-dependent consumption of GSSG reaching the plateau after 10 min of incubation (data not shown). The GSSG consumption was accompanied by a stoichiometric formation of GSH and GSH-NAC disulfide (data not shown), thus confirming the disulfide breaking activity according to the following equilibrium:

GSSG + NAC = GSH + GSH-NAC(1)

The calculated K_app_ of NAC was 0.24 M^−1^ s^−1^ and that of Cys of 1.99 M^−1^ s^−1^. The greater reactivity of Cys is well explained by considering its higher acidity with respect to NAC (pKa NAC = 9.7 vs. 8.4 of Cys) [20]. The pH-independent rate constant of the thiolate form of Cys and NAC toward GSSG were calculates as logK_NAC_ = 1.69; logK_Cys_ = 1.29, respectively, and this greater nucleophilicity of the NAC thiolate in respect to that of Cys is due to the reduced acidity of the thiol as already described for other thiols [21].

### 3.2. Albumin Cys34-Cys Breaking Activity

**Figure 1:**
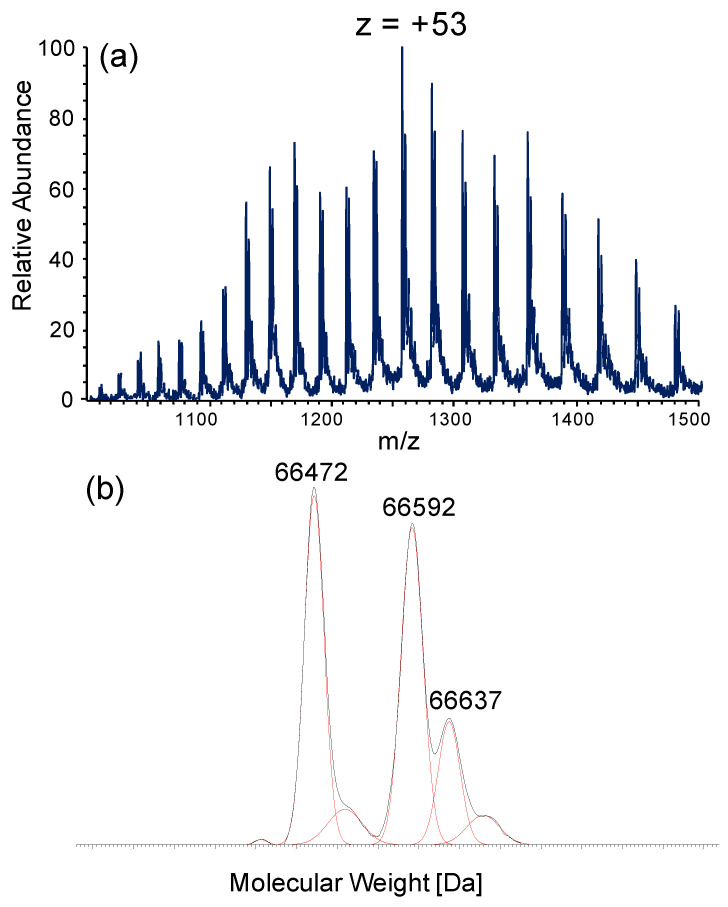
ESI-MS spectrum of intact HSA: (**a**) Multicharged ion profile of HSA. (**b**) Deconvoluted spectrum of HSA spectrum showing the mercaptoalbumin (HSA-SH) isoform at 66472 Da, the cysteinylated form, HSA-Cys, at 66592 Da (+120 Da with respect to HSA-SH) and the glycated form, HSA-gly, at 66637 Da (+165 Da).

The effect of NAC and Cys in breaking the Cys34-Cys disulfide was then determined in human plasma, provided by Sigma, by measuring the relative content of mercaptoalbumin and of the corresponding cysteinylated form by intact protein analysis. As shown in Figure 1, the ESI-MS spectrum of intact HSA shows a multicharged ions profile and the deconvoluted spectrum reports three main isoforms at 66472, 66592, 66637 Da, relative to the mercaptoalbumin (HSA-SH), the cysteinylated (HSA-Cys, +120 Da) and the glycated forms (HSA-gly; +165 Da). The relative abundance of the three isoforms was calculated as follows: HSA-SH: 40%, HSA-Cys: 32%, HSA-gly: 12%.

**Figure 2:**
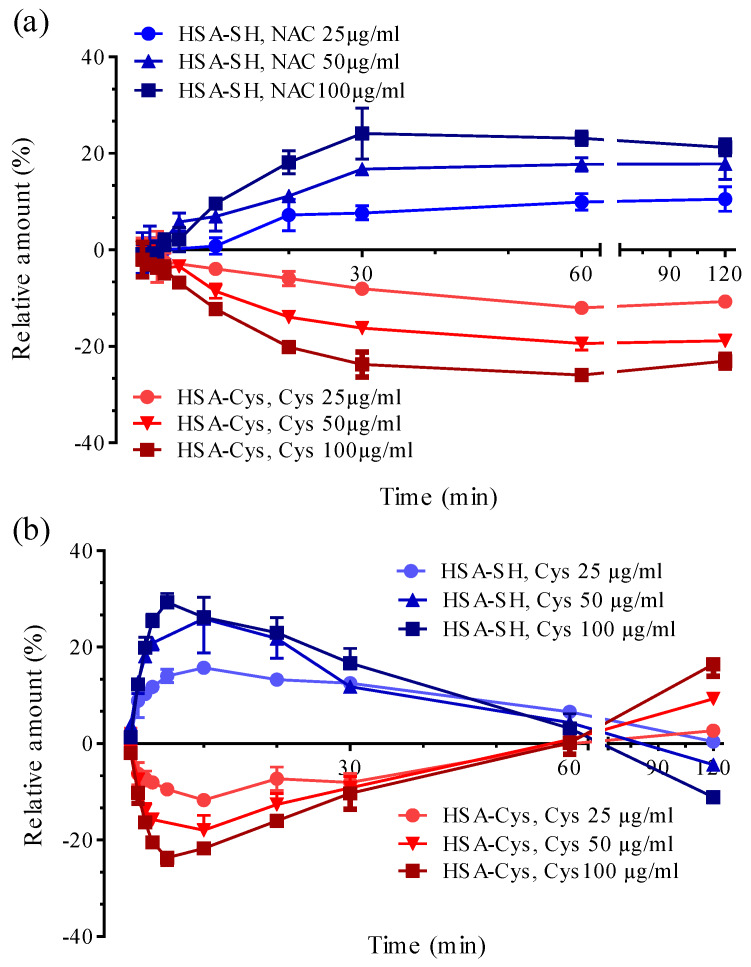
HSA-Cys breaking activity of NAC and Cys: Time- and dose-dependent effect of NAC (**a**) and Cys (**b**) in restoring mercaptoalbumin (upper part of the graph) by reducing the content of the cysteinylated form (lower part of the graph). Legends to the symbol are reported in the figure.

Figure 2 shows the time- and dose-dependent effect of Cys (panel a) and NAC (panel b) in restoring mercaptoalbumin (upper part of the graph) by reducing the content of the Cys34 form (lower part of the graph) through a disulfide breaking of the Cys34-Cys bond. In the graph, the relative content of both HSA-SH and HSA-Cys at the beginning of the kinetic are set as 0 value. 

The strict correlation also in quantitative terms between HSA-SH increase and HSA-Cys consumption indicates that the disulfide breaking effect of NAC regenerates Cys34 by forming the mixed disulfide NAC-Cys according to the following equilibrium:

HSA-Cys + NAC = HSA-SH + Cys-NAC(2)

The formation of Cys-NAC/HSA-SH and not of HSA-NAC/Cys-Cys is explained by considering that the entering nucleophilic moiety (the NAC thiolate) breaks the disulfide bonds and remains linked to the more basic sulfur atom (Cys) so displacing the more acid thiol function of Cys34. 

The quantitative correspondence between the amount of regenerated Cys34 and the consumed cysteinylated form also well confirms that the disulfide breaking activity of NAC is specific for Cys34-Cys and does not affect the other disulfides of albumin [22]. 

In our experimental conditions the formation of HSA adducted by NAC was not detected at least within the maximum incubation time of 120 min and this is in accordance with Harada who detected the NAC adduct with HSA only after 6 hours of incubation [10] which is a quite extensive incubation time considering that the T_1/2_ of NAC corresponds to 3.97 hours after an oral intake. We selected a maximum incubation time of 2 hours corresponding to the T_max_ of NAC [23]. 

**Figure 3:**
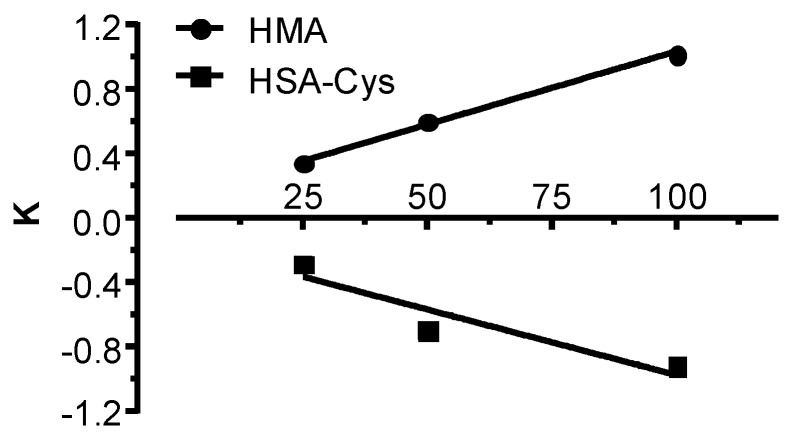
Regeneration effect of HSA-SH by NAC: Dose-dependent effect of NAC in regenerating HSA-SH (positive slope) and in breaking (negative slope) the cysteinylated form.

The effect of NAC was time-dependent, reaching the maximum regenerating effect after 30 min of incubation. As shown in Figure 3, the dose-dependent breaking effect was linear within a concentration range of 25–100 µg/mL. The slope of 0.009 for the HSA-SH regeneration almost overlaps the slope of −0.008 for HSA-Cys consumption, confirming the reaction (2).

According to the reaction constant as determined in the previous paragraph, the breaking effect of Cys was more rapid, reaching the plateau of HSA-SH formation after only 5 min. As observed for NAC, also in the case of Cys, a clear correlation between HSA-SH formation and HSA-Cys reduction was observed. However, in contrast to what was observed for NAC, in the case of Cys we found that immediately after the maximum recovery of HSA-SH, its relative content started to reduce in parallel to the formation of the cysteinylated form. After almost 60 min of incubation the complete recovery of HSA-SH was abolished and HSA-Cys reached the initial concentration. After this time the cysteinylated form of HSA further increased with respect to the initial content, reaching at the highest concentration a relative content of HSA-SH and HSA-Cys of 40.38% and 30.46% (with NAC: 100 µg/mL), respectively. Based on this data, it is clear that in the case of Cys, the dimer Cys-Cys rapidly reacts with Cys34 forming again the mixed disulfide HSA-Cys. This behavior was not observed in the presence of NAC since the relative content of Cys34 was maintained after reaching the maximum amount of recovery, to indicate that the disulfide NAC-Cys is stable and does not react with Cys34 to form the cysteinylated derivative. 

To better understand the different reactivity of the disulfides Cys-Cys and NAC-Cys toward Cys34 a molecular modeling study was then carried out as reported in Section 3.4.

### 3.3. HSA-Cys Breaking Activity of NAC and Cys in Human Plasma from Healthy Donors

The next step was to confirm the HSA-Cys breaking activity of NAC in fresh human plasma collected from healthy donors and characterized by a less content of cysteinylated albumin with respect to that contained in the pool of plasma from Sigma. A concentration range of NAC (1–50 µg/mL) which can be achieved after os or iv NAC treatment was used. Nolin et al. reported a Cmax (maximum plasma concentration) of 2.5 µg/mL after a single 600 mg oral dose which increases to 10 µg/mL in end-stage renal disease patients and doubling to 20 µg/mL after a 1200 mg dose [23]. Plasma NAC concentration after an Iv infusion ranges between 20 and 40 µg/mL as reported by Soldini et al. [24].

**Figure 4:**
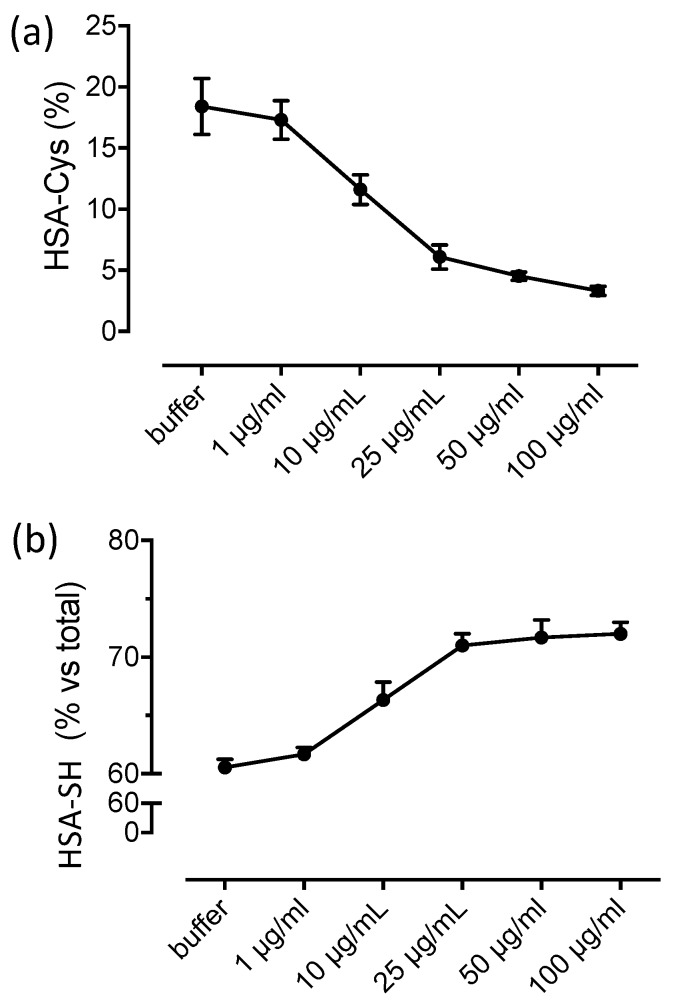
NAC regenerates HSA-SH in plasma of healthy donors: Dose-dependent effect of NAC in regenerating HSA-SH (**a**) by breaking the cysteinylated form (**b**) in human plasma of healthy donors.

Figure 4 shows the dose-dependent effect of NAC in terms of HSA-SH regeneration and HSA-Cys breaking activity after 60 min of incubation. The relative content of HSA-SH and HSA-Cys in absence of NAC were 84% and 10% while the glycated form was around 5%. The breaking activity of NAC was already observable at 1 µg/mL, to induce an almost complete recovery of HSA-SH and a concomitant disappearance of HSA-Cys at 25 µg/mL.

**Figure 5:**
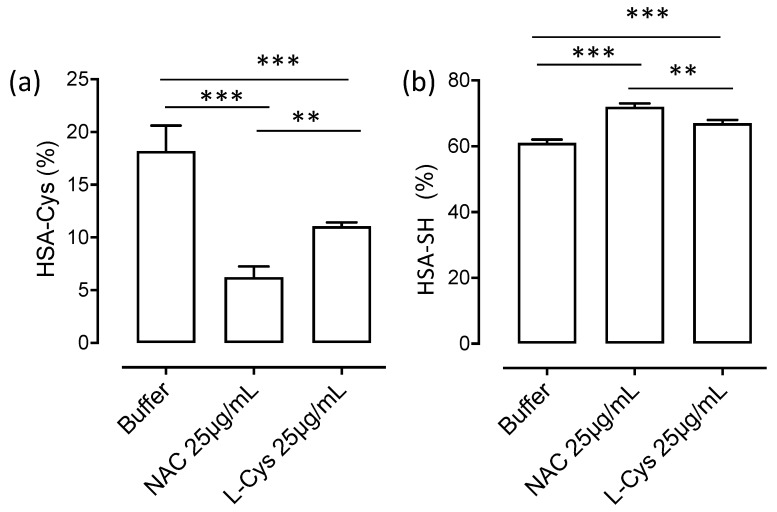
Comparative analysis of NAC and Cys in breaking HSA-Cys (**a**) and restoring HSA-SH (**b**). NAC and Cys were incubated with human plasma for 60 min at 37 °C. Significant differences are indicated by * *p* < 0.001 and ** *p* < 0.001; ANOVA test.

The breaking activity of NAC was then compared to that of Cys at a fixed dose of 25 µg/mL. As shown in Figure 5, NAC was more effective in both restoring HSA-SH and in reducing HSA-Cys and this is well explained by the fact that even if Cys is more reactive, its efficacy is greatly reduced by the reactivity of Cys-Cys disulfide with Cys34 that after one hour of incubation greatly reduced the regenerating activity. 

### 3.4. Molecular Modeling Studies

**Figure 6:**
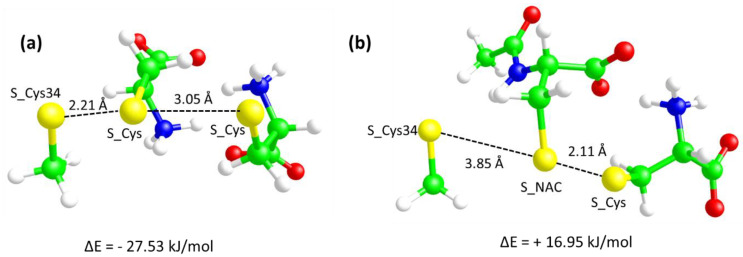
Molecular Modeling studies: Structures of the two studied trisulfide intermediates-Cys-Cys-C34 (**a**) and Cys-Nac-C34 (**b**) as optimized by DFT calculations by simulating the water effect using the PCM method. The relative energies with respect to the isolated reactants are also reported.

With a view to explaining the different capacities of Cys and NAC to yield cysteinylated adducts, the nucleophilic exchange between the possible disulfides (Cys-Cys and Cys-NAC) and Cys34 (represented by methanethiol for simplicity) to generate Cys-Cys34 and NAC-Cys34, respectively, were simulated at gas phase and in water using the PCM method by density functional theory (DFT) using the Becke three-parameter hybrid function with LYP correlation (DFT/B3LYP) and with the 6-31G basis set as implemented by the GAMESS software [19]. For simplicity Cys34 was represented by a methanethiol molecule which in fact corresponds to the side-chain of the cysteine. Such approximation is justified by the clear impossibility to account for the protein environment by DFT calculations and on the consideration that the reactivity of the thiolate moiety is unaffected by the Cys34 backbone atoms. In detail, the simulations were focused on the formation of the key anionic trisulfide intermediates. Figure 6 compares the two minimized intermediates and allows for some key observations. The first consideration concerns the optimized distances between the sulfur atoms.

Indeed, and while in the starting structures of both intermediates the three sulfur atoms were placed so as to be equidistant, with the S-S distances equal to 2.2 Å, the optimized structures show differences which are suggestive of their different reactivity. In detail, Figure 6, panel a shows that in the trisulfide intermediate involving the Cys-Cys reactant, the S–S distance between Cys-S-S-Cys34 remains at a distance comparable to the initial one (2.21 Å), while the Cys-S-S-Cys distance progressively increases up to an optimized value of 3.06 Å. Such a final structure is suggestive of a breaking of the starting Cys-Cys disulfide with the corresponding exchange with Cys34. In contrast, the optimized trisulfide intermediate involving the Cys-NAC reactant (Figure 6, panel b) reveals an opposite situation since the final Cys-S-S-NAC distance remains equal to 2.11 Å, while the Cys34-S-S-NAC distance progressively increases up to an optimized value of 3.85 Å. The different behavior is also confirmed by the angle values between the sulfur atoms (as defined by CH_2_-S^….^S atoms). Indeed, while in the Cys-Cys-C34 system all three angles assume values (around 100°) which are suggestive of the observed nucleophilic exchange, in the Cys-NAC-C34, the Cys 34 S atom approaches the S atom of NAC with an unsuitable orientation (around 90°) which confirms its incapacity to assume a pose conducive to the exchange. The optimized structure depicted in Figure 6, panel b indicates therefore that the Cys-NAC disulfide is unable to exchange with Cys34. This different reactivity finds compelling confirmation in the relative energies of the intermediates with respect to the isolated reactants as computed by DFT calculations accounting for the water solvent by using the PCM approach. Indeed, the negative relative energy of the intermediate involving the Cys-Cys disulfide is deep enough to allow for the corresponding nucleophilic exchange, while the positive energy value for the intermediate with the Cys-NAC disulfide clearly reveals that the corresponding exchange cannot occur. 

A second observation concerns the salt bridges characterizing the starting disulfides and the corresponding intermediates. In detail, the Cys-Cys disulfide is a neutral zwitterionic molecule and its structure is stabilized by two ion-pairs between the corresponding charged termini, while the Cys-NAC disulfide is an anionic molecule, the structure of which involves only one ion-pair since the carboxylated of the Cys residue remains uncoupled due to the acetyl group of NAC. Clearly, this difference influences the approach of the anionic Cys34-S-moiety. Concerning the reaction involving the Cys-Cys disulfide, Figure 6a shows that an ammonium head is addressed toward the sulfur atoms favoring both the approach of Cys34-S- and the resulting exchange. By contrast, the negative charge of the Cys-NAC disulfide prevents the approach of Cys34-S- and destabilizes the corresponding intermediate due to simple electrostatic repulsion. 

When simulating the formation of the trisulfide intermediates in gas phase, the difference between the two relative energies markedly increases (ΔE = −243.81 kJ/mol and ΔE = +120.46 kJ/mol) for the intermediates involving Cys-Cys and Cys-NAC, respectively. This finding appears to be particularly interesting for the nucleophilic exchange involving the Cys-Cys disulfide which appears greatly favored when removing the solvent effect. On one hand, this result can be explained by considering the shielding effect of water molecules on the ion-pairs promoting the reaction (see above), on the other hand this suggests that such a nucleophilic exchange would occur more favorably when involving cysteine residues embedded within rather hydrophobic environments as in the case of Cys34 which is inserted into an apolar crevice. 

### 3.5. Antioxidant Activity

The antioxidant activity was tested using the total peroxyl radical-trapping potential (TRAP) assay that is based on a non-fluorescent substrate, which is oxidized to the fluorescent oxidation product by the radical initiator AAPH which generates a constant flux of radical species. The antioxidant activity is measured by calculating the lag-phase induced before the substrate oxidation [16,25]. 

**Figure 7:**
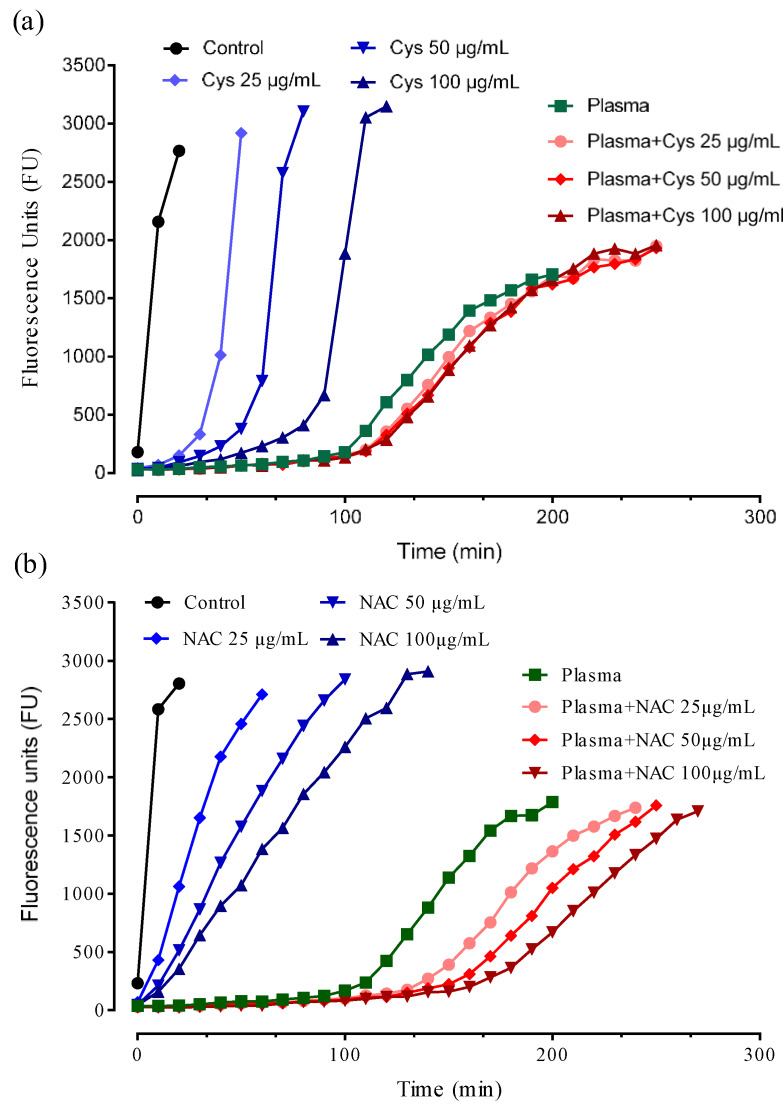
TRAP assay: Antioxidant activity of Cys (**a**) and NAC (**b**) in absence (filled symbols) and presence (empty symbols) of human plasma.

Figure 7 shows the antioxidant activity of NAC and Cys (empty symbols) in a concentration range of 25–100 µg/mL.

**Figure 8:**
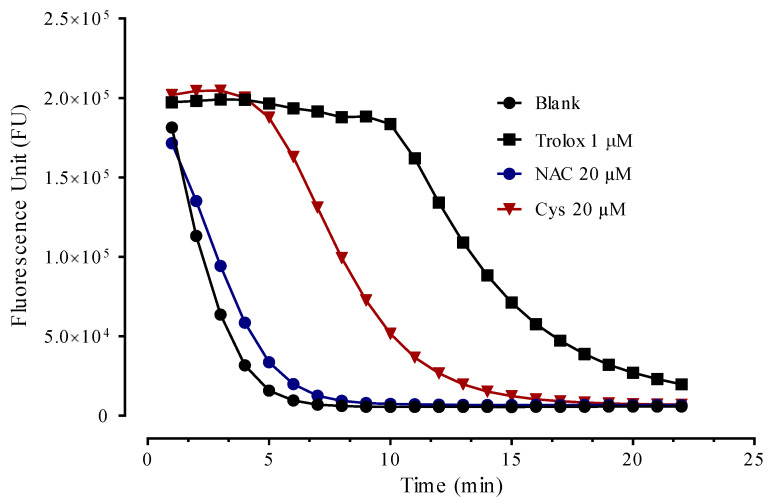
ORAC assay: radical scavenging of Cys, NAC and Trolox.

NAC was unable to induce a lag-phase even at the maximum dose used while a slight and dose-dependent reduction of the rate of DCFH oxidation was observed indicating a slight antioxidant activity but not enough to totally protect the substrate from the oxidation. In contrast, Cys induced a dose-dependent lag-phase due to its greater direct antioxidant activity with respect to NAC, as expected. The greater direct antioxidant of Cys with respect to NAC was then confirmed by using the ORAC assay in which the antioxidant activity of NAC and Cys is compared to that of Trolox (Figure 8). 

Human plasma induced a significant lag-phase which is the result of the combination of the several plasma antioxidants including low molecular species, mainly uric acid, and proteins. Several papers investigated the contribution of low and large molecules in plasma/serum to the total antioxidant capacity. Wayner et al. first used the AAPH oxidation system to quantitatively evaluate the antioxidant potential of human plasma and even if uric acid and vitamin E were identified as important players, concluded that up to 73% of plasma antioxidant activity was associated with “protein and lipoprotein material” [26].

More recently, Swertfeger et al. [27] using a proteomic and selective precipitation method, identified the major antioxidant contributors able in preventing low-density lipoprotein (LDL) oxidative modification, which is one factor thought to initiate and propagate plaque development, according to the “oxidative modification hypothesis” [28]. The results indicated that ≈15% of activity is contributed by fibrinogen, ≈20% by IgG, ≈1%–2% by HDL and ≈6% by small molecules, being albumin the main antioxidant accounting for ≈40% of the antioxidant activity against water soluble radical and copper-initiated oxidation mechanisms. Furthermore, it was reported that more than 70% of the free radical-trapping activity of serum is due to HSA as assayed by the free radical-induced hemolysis test [29]. These results indicate that HSA represents the major and predominant antioxidant in plasma whose relative contribution was reported to range from 40% to 70% of the total antioxidant capacity of plasma.

The antioxidant activity of albumin can be referred to Cys34 as well to the six methionine (Met) residues and whose contribution was investigated using recombinant HSA mutants, in which Cys34 and/or the six Met residues had been mutated to Ala [30]. The results indicate that Cys34 plays the most important role for the antioxidant activity of HSA against the tested oxidant species, namely O_2_^•−^, ^•^NO, HO^•^, HOCl, and H_2_O_2_. 

Taking together, the data from the literature well indicate that HSA accounts from 40% to 70% of the total antioxidant capacity of plasma which is mainly due to the presence of Cys34. As above discussed, when Cys34 acts as antioxidant, it is converted to the cysteinylated form to prevent a further oxidation to the irreversible forms. In the cysteinylated form, Cys34 is clearly not effective as antioxidant, making the ability of NAC to selectively restore Cys34 a potential mechanism of restoring the antioxidant capacity of plasma in oxidative stress conditions. 

**Figure 9:**
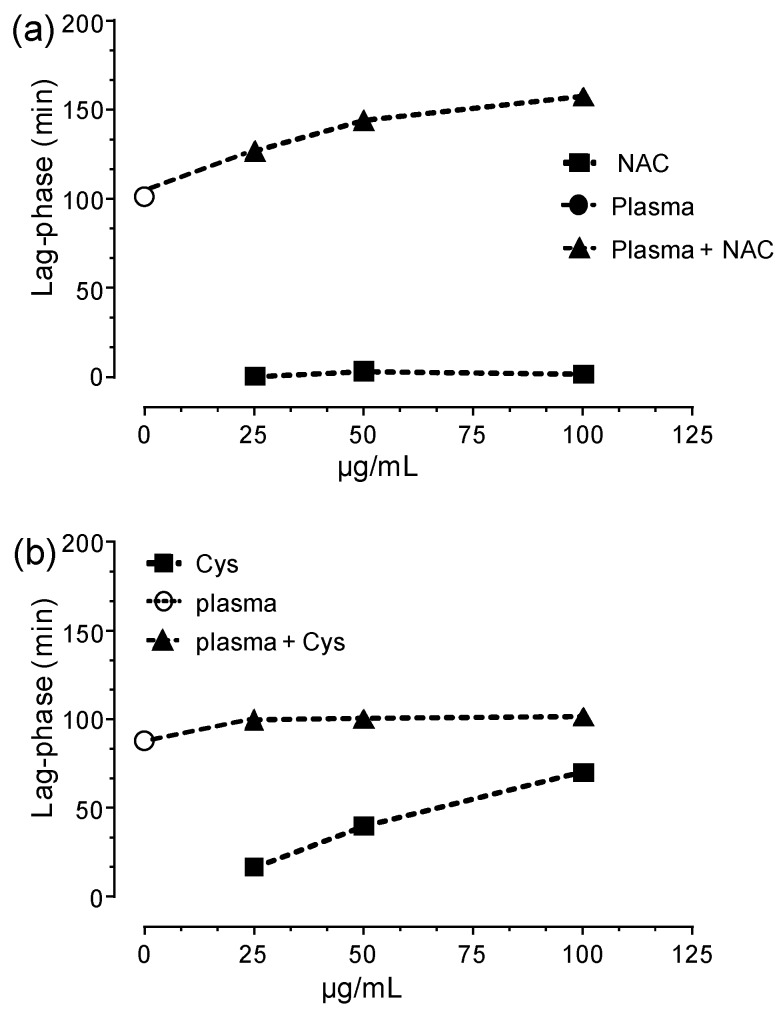
Dose-dependent lag-phase induced by NAC (**a**) and Cys (**b**) in absence and presence of plasma.

To demonstrate this important mechanism, human plasma was incubated with NAC and then oxidized by the radical initiator. As shown in Figure 7, NAC dose-dependently increased the plasma antioxidant activity. Figure 9 shows the relationship between dose and lag-phase for NAC in absence and presence of plasma. The lag-phase induced by plasma accounted for almost 100 min which increased by NAC spiking by 27%, 45%, and 60% at the doses of 25, 50 and 100 µg/mL, respectively.

It should be underlined that NAC alone, at the same doses, was found unable to induce the lag-phase (Figure 8 and Figure 9) thus demonstrating that the effect of NAC in plasma is not due to a direct effect but rather to an indirect effect mechanism mediated by the Cys34 regeneration. Although Cys as such dose-dependently increased the lag-phase, when it was spiked to plasma it did not significantly increased the lag-phase (Figure 8 and Figure 9). This effect is explained by considering the kinetic as reported in Section 3.2. In particular, during the incubation of Cys with plasma, it rapidly breaks the disulfide bond and free Cys34 but then the reverse reaction mediated by Cys-Cys (also formed by the oxidation of Cys in plasma) reforms the cysteinylated form of Cys34 thus neutralizing the antioxidant effect.

### 3.6. NAC Is an Indirect Antioxidant at Extracellular Level: The Role of Cys34

**Figure 10:**
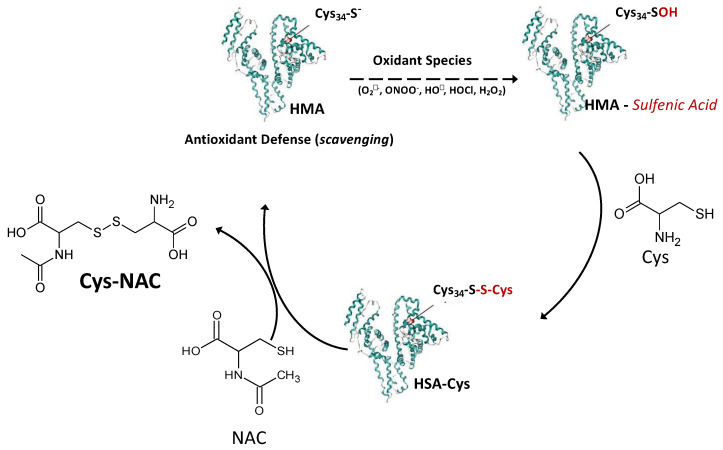
Indirect antioxidant mechanism of NAC at extracellular level - Cys34 of mercaptoalbumin (HSA-SH) efficiently scavenges oxidant species forming the corresponding sulfenic acid which then reacts with Cys to form the cysteinylated adduct (HSA-Cys). NAC is able to selectively break the disulfide bond of HSA-Cys leading the disulfide NAC-Cys and the free form of Cys34 which in turn further acts as antioxidant.

The overall data well indicates that NAC is able to restore the antioxidant activity of plasma even if the reaction constants of NAC toward the most reactive oxidizing species do not make NAC a direct scavenger. The mechanism is summarized in Figure 10 where Cys34 of albumin acts with a pivotal role in the antioxidant activity of plasma. 

When Cys34 scavenges the oxidant species, the corresponding sulfenic acid is formed which then reacts with Cys to form the cysteinylated adduct. NAC is able to selectively break the disulfide bond leading the disulfide NAC-Cys and the free form of Cys34 which in turn further acts as antioxidant. In contrast to other disulfides, such as Cys-Cys, NAC-Cys does not react back with Cys34 as demonstrated by molecular modeling studies, thus maintaining the Cys34 in a free form, making NAC a suitable thiol-disulfide breaking compound.

## 4. Conclusions

In conclusion, the present paper demonstrates that NAC enhances the plasma antioxidant activity by restoring Cys34 through a selective thiol-disulfide mechanism. The ability of NAC to restore Cys34 in patients with oxidative-based diseases and characterized by an impaired balance between Cys34 in free and disulfide form is currently under investigation by us. By considering the role of oxidative stress and in particular of plasma oxidation in several diseases, the availability of a drug able to regenerate the endogenous defense is of great value and its application to chronic diseases, where oxidative stress plays an important role, should be carefully evaluated. Finally, it should be considered that NAC restores albumin Cys34 which is an endogenous protein with a half-time of three weeks and hence the antioxidant activity of NAC is not strictly related to its residence time (short due to its rapid clearance) as it would be in the case of a direct exogenous antioxidant.
